# Effect of Aluminum Adjuvant and Preservatives on Structural Integrity and Physicochemical Stability Profiles of Three Recombinant Subunit Rotavirus Vaccine Antigens

**DOI:** 10.1016/j.xphs.2019.10.004

**Published:** 2020-01

**Authors:** Sanjeev Agarwal, John M. Hickey, David McAdams, Jessica A. White, Robert Sitrin, Lakshmi Khandke, Stanley Cryz, Sangeeta B. Joshi, David B. Volkin

**Affiliations:** 1 Department of Pharmaceutical Chemistry, Vaccine Analytics and Formulation Center, University of Kansas, Lawrence, Kansas 66047; 2 The Center for Vaccine Innovation and Access, PATH, Seattle, Washington 98121; 3 The Center for Vaccine Innovation and Access, PATH, Washington, District of Columbia 20001

**Keywords:** rotavirus, vaccine, adjuvant, formulation, stability, thimerosal, preservative

## Abstract

A nonreplicating rotavirus vaccine (NRRV) containing 3 recombinant fusion proteins adsorbed to aluminum adjuvant (Alhydrogel [AH]) is currently in clinical trials. The compatibility and stability of monovalent NRRV antigen with key components of a multidose vaccine formulation were examined using physicochemical and immunochemical methods. The extent and strength of antigen-adjuvant binding were diminished by increasing phosphate concentration, and acceptable levels were identified along with alternate buffering agents. Addition of the preservative thimerosal destabilized AH-adsorbed P2-VP8-P[8] as measured by differential scanning calorimetry. Over 3 months at 4°C, AH-adsorbed P2-VP8-P[8] was stable, whereas at 25°C and 37°C, instability was observed which was greatly accelerated by thimerosal addition. Loss of antibody binding (enzyme-linked immunosorbent assay) correlated with loss of structural integrity (differential scanning calorimetry, fluorescence spectroscopy) with concomitant nonnative disulfide bond formation (sodium dodecyl sulfate-polyacrylamide gel electrophoresis) and Asn deamidation (liquid chromatography -mass spectrometry peptide mapping). An alternative preservative (2-phenoxyethanol) showed similar antigen destabilization. Due to limited availability, only key assays were performed with monovalent P2-VP8-P[4] and P2-VP8-P[6] AH-adsorbed antigens, and varying levels of preservative incompatibility were observed. In summary, monovalent AH-adsorbed NRRV antigens stored at 4°C showed good stability without preservatives; however, future formulation development efforts are required to prepare a stable, preservative-containing, multidose NRRV formulation.

## Introduction

Rotavirus (RV) is a leading cause of childhood gastroenteritis and severe diarrhea worldwide with ∼128,500 deaths (children below 5 years of age) in 2016.^1^ The majority of RV-related mortality occurs in developing countries where rehydration therapy is more difficult to obtain and efficacy of currently available live-attenuated, oral RV vaccines is lower compared with that in the developed world.^2^ Reasons behind the lower efficacy are multifactorial and not well understood.^3^ There is thus a great need for a new-generation RV vaccine with enhanced efficacy for the developing world at affordable costs with consistent supply to improve vaccine coverage.^4^ A trivalent subunit RV vaccine (nonreplicating rotavirus vaccine [NRRV]) contains 3 recombinant protein antigens (belonging to RV genotypes P[4], P[6], and P[8]; see next paragraph) and is currently in clinical trials in South Africa.^5^ These 3 antigens provide broad worldwide serotype coverage to RV infections with the P[6] genotype much more prevalent in the African and Southeast Asian regions.^6^ Previously, a monovalent NRRV adsorbed to Alhydrogel (AH) was shown to be safe and immunogenic in infants and toddlers in early-stage clinical trials.^7^

Each of the 3 NRRV protein antigens is a recombinant fusion of the truncated VP8* protein (a cleavage product of the RV outer capsid protein VP4) linked to CD4^+^ T cell epitope (P2) from tetanus toxoid using a GSGSG linker.^8^^,^^9^ The 3 antigens are named P2-VP8-P[4], P2-VP8-P[6], and P2-VP8-P[8] where P2 is the tetanus toxoid epitope, VP8 the truncated VP8* protein, and P[x] denotes the P genotype from the human RV strains DS-1-like (G2P[4]), 1076-like (G2P[6]), and Wa-like (G1P[8]). The 3 NRRV antigens are referred to as P[4], P[6], and P[8] in this work. Wen et al.^9^ showed that addition of an aluminum-based adjuvant to P2-VP8-P[6/8] containing monovalent vaccines increased the neutralizing antibody titers against RV in guinea pigs; however, the binding of antigens to adjuvant (aluminum phosphate) was not taken into consideration. The clinical NRRV is formulated with AH to keep the NRRV antigens bound to adjuvant.^7^^,^^10^

Affordability and accessibility are major hurdles during successful incorporation of a new vaccine in the national immunization programs of developing countries.^4^ Using multidose formulations is an effective strategy to reduce the cost of vaccine per dose in terms of manufacturing, packaging, storage, transport, and medical waste.^11^ However, the suitability of a multidose formulation strategy is vaccine specific depending on inherent cost, patient demand, and vaccine wastage.^12^ Because there is potential risk of microbial contamination due to multiple withdrawals from the same vial, a multidose vaccine drug product requires the addition of a preservative.^13^ Combining multiple vaccines into a single vial (combination vaccines) also provides many economic benefits such as fewer vaccination visits and reduced manufacturing costs (fewer doses, less packaging, and streamlined storage and handling). In addition, societal benefits of combination vaccines include improved vaccine coverage, fewer missed or delayed vaccinations, and fewer needle stick injuries.^14^^,^^15^

The long-term goal of NRRV development is to introduce the NRRV into the pentavalent childhood combination vaccine (e.g., pentavalent diphtheria, tetanus, whole-cell pertussis [wcP], Hib, and HepB) to lower costs, enhance patient compliance, and improve RV vaccine coverage. Pediatric combination vaccines in the developing world typically contain wcP and thimerosal,^16^ with the latter used both as a preservative and inactivating agent during wcP production. Therefore, it is important to assess the compatibility and stability of NRRV antigens with thimerosal, an ethyl mercury containing compound that has been used in multidose injectable vaccines since 1930s to protect against potential microbial contamination. The addition of thimerosal to certain vaccines, for example, HPV and IPV, is known however to reduce their *in vitro* potency and *in vivo* immunogenicity.^17^^,^^18^

In this work, we evaluated the binding of NRRV antigens to AH in the presence of various buffering agents (i.e., histidine, HEPES, Tris, phosphate) to ensure complete protein binding while maintaining good buffering capacity. The adsorptive capacity and strength of the P[8] antigen binding to AH was determined along with the protein’s structural integrity, physicochemical stability, and antibody binding during storage stability studies (± the preservatives thimerosal and 2-phenoxyethanol [2-PE]). The physicochemical stability of the NRRV antigens on the surface of the AH was examined by a combination of immunochemical (enzyme-linked immunosorbent assay [ELISA]), biochemical (sodium dodecyl sulfate-polyacrylamide gel electrophoresis [SDS-PAGE] combined with liquid chromatography-mass spectrometry (LC-MS) peptide mapping), and biophysical (differential scanning calorimetry [DSC], fluorescence spectroscopy) methods. Due to limited availability of P[4] and P[6] antigens, a subset of key results obtained with P[8] were assessed with these 2 aluminum-adsorbed NRRV antigens. These results are discussed in the context of future formulation development efforts to be undertaken to develop a more stable, preservative-containing, multidose formulation of the trivalent NRRV.

## Materials and Methods

The NRRV antigens (P[4], P[6], and P[8]) were produced and purified from *E. coli* at Blue Sky BioServices, MA, and provided frozen in 600 mM ammonium sulfate, 50 mM Tris buffer at pH 7.5. AH adjuvant was purchased from Accurate Chemical Scientific Corporation (Westbury, NY). Sodium chloride and sodium phosphate dibasic heptahydrate were purchased from Thermo Fisher Scientific (Waltham, MA). Sodium phosphate monobasic monohydrate, Histidine, HEPES, Tris, and all other reagents and chemicals were purchased from Sigma-Aldrich (St. Louis, MO) and were of analytical grade or higher. Extinction coefficient used for concentration determination of each antigen has been reported previously.^19^

Experimental details including sample preparation and setup of storage stability studies are provided in the Supplemental Methods section. Details of several of the methods used in this work have been described previously,^19^^,^^20^ and the experimental setups and analytical methods used are provided in the Supplemental Methods section including zeta potential (ZP), antigen-adjuvant binding, Langmuir binding isotherm construction, intrinsic Trp fluorescence spectroscopy (steady state and time-resolved), SDS-PAGE analysis coupled with LC-MS peptide mapping, and antibody binding as measured by an inhibition ELISA.

## Results

### P[8] Antigen-AH Adjuvant Interactions

**Figure 1:**
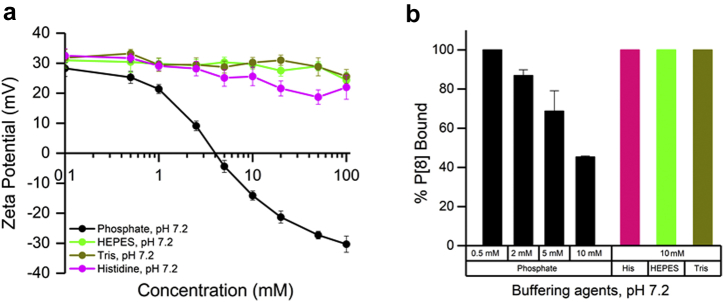
Surface charge and percent P[8] antigen binding to Alhydrogel (AH) in different buffers. (a) Zeta potential of AH at pH 7.2 after pretreatment with increasing concentrations of different buffering agents. (b) Percent P[8] bound to AH at pH 7.2 in different buffering agents (60 μg P[8] antigen added to 0.56 mg of aluminum as AH in the presence of 0.15 M NaCl at pH 7.2). Error bars represent the range from duplicate measurements.

The pretreatment of AH with phosphate ions is known to lower its net surface charge due to replacement of hydroxyl groups on AH with phosphate ions.^21^ In this work, as expected, pretreatment of AH with increasing concentrations of phosphate buffer at pH 7.2 altered the ZP values of the AH adjuvant from positive to negative. The ZP values of AH did not change substantially (positive ZP > 20 mV) when incubated with up to 100 mM HEPES, Tris, or histidine buffers at pH 7.2 (Fig. 1a). Similar results were obtained with AH and 100 mM histidine at pH 6.5 or 6.8 (data not shown). As shown in Figure 1b, 100% P[8] antigen was bound to AH in the presence of 0.5 mM phosphate, 10 mM histidine, 10 mM Tris, or 10 mM HEPES. A decreasing trend was observed in antigen binding with the addition of higher concentrations of phosphate (e.g., ∼40% of P[8] bound in 10 mM phosphate, Fig. 1b) as the surface charge of AH became negative (as measured by ZP values).

**Figure 2:**
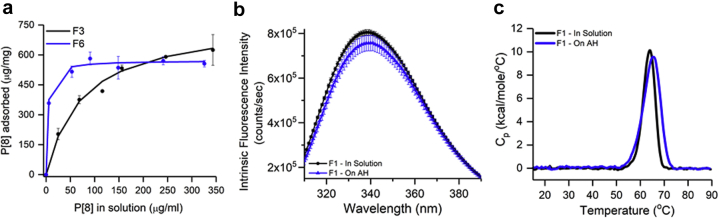
P[8] antigen interactions with the aluminum-adjuvant Alhydrogel (AH) in different formulations. (a) Langmuir-binding isotherms of P[8] antigen with AH in formulations F3 and F6. (b) Tertiary structure integrity analysis of P[8] using intrinsic tryptophan fluorescence emission spectra at 10°C in solution (black) and bound to AH (blue) in F1 (see Supplementary Fig. S2 for data in F3 and F5 formulations). (c) Conformational stability analysis of P[8] with representative DSC thermograms in solution (black) and bound to AH (blue) in formulation F1 (see Sup. Supplementary Table S1 for T_onset_. T_m_ and ΔH’ values). See Table 1 for composition of the different formulations. Error bars represent 1 SD from triplicate measurements.

Langmuir binding isotherms were generated in 6 different formulations (see Table 1 for composition), at pH 7.2 or 6.8, to determine the adsorptive strength and capacity values of P[8]-AH interactions. As shown in Figure 2a and Table 1, the adsorptive strength values of P[8]-AH interactions were substantially lower in phosphate formulations (F1, F3) versus nonphosphate containing formulations (F5, F6, F7, F8). The adsorptive capacity in each formulation, however, was still substantially higher than the targeted clinical doses (i.e., up to 60 μg of each NRRV antigen per 0.56 mg of aluminum).

**Table 1:** Summary of NRRV Antigen (P[8], P[6], and P[4]) and Aluminum Adjuvant (Alhydrogel, AH) Binding Parameters and Composition of the Different Formulations

NRRV Antigen	Formulation	Adsorptive Strength (mL/mg)	Adsorptive Capacity (μg/mg)	R^2^
P[8]	F1	13 ± 5	1000 ± 0	0.97
	F3	14 ± 4	774 ± 119	0.98
	F5	112 ± 77	718 ± 103	0.99
	F6	338 ± 96	572 ± 33	0.99
	F7	105 ± 89	607 ± 37	0.99
	F8	87 ± 87	750 ± 167	0.99
	F1 + TH	10 ± 2	839 ± 140	0.97
	F3 + TH	54 ± 23	670 ± 89	0.95
	F5 + TH	81 ± 67	611 ± 111	0.96
P[6]	F1	7 ± 5	940 ± 342	0.97
	F3	10 ± 2	646 ± 42	0.90
	F5	66 ± 55	917 ± 167	0.97
P[4]	F1	11 ± 7	749 ± 321	0.94
	F3	10 ± 0	742 ± 55	0.92
	F5	346 ± 120	750 ± 167	0.99
Formulation Composition				

Formulation #	Buffer	Salt (NaCl)	PS-80 (w/v)	pH
F1	0.5 mM Sodium phosphate	0.15 M	–	7.2
F2	0.5 mM Sodium phosphate	0.15 M	0.006%	7.2
F3	0.5 mM Sodium phosphate	0.15 M	0.025%	7.2
F4	5 mM Histidine	0.15 M	0.006%	6.8
F5	5 mM Histidine	0.15 M	0.025%	6.8
F6	5 mM Histidine	0.15 M	0.025%	7.2
F7	5 mM HEPES	0.15 M	0.025%	7.2
F8	5 mM Tris	0.15 M	0.025%	7.2

Antigen-adjuvant binding data were fitted to linear form of Langmuir adsorption equation as described in Methods. See Figure 2a for representative binding isotherms with P[8]. Values represent mean ± range from 2 measurements. Second part of the table describes the composition of the different formulations (F1-F8) described in this work.

TH, 0.01% Thimerosal.

The structural integrity of solution versus AH-bound P[8] was assessed by intrinsic Trp fluorescence spectroscopy and DSC. As shown in Figure 2b, the intrinsic Trp fluorescence emission spectra of P[8] at 10°C in F1 are overall similar, demonstrating overall similar microenvironments for the average Trp residues of P[8] when bound to AH (albeit a small red shift of ∼1-2 nm in the lambda-max peak position for P[8] was noted when bound to AH compared with solution, potentially indicating very subtle structural differences). On heating, solution versus AH-bound P[8] showed similar thermal onset values, albeit the subsequent thermal transitions differed in terms of extent and direction of intensity changes, likely due to the P[8] protein aggregating in solution versus on the AH surface (Supplementary Fig. S2-A2), across various formulations (no effect of buffer type or 0.025% PS-80). The conformational stability of solution versus AH-bound P[8] showed similar thermal melting values (T_m_) and apparent enthalpy (ΔH’) of unfolding values by DSC (Fig. 2c), although the T_onset_ value decreased by ∼1.5°C, the T_m_ value was ∼1°C higher and the apparent enthalpy (ΔH’) was ∼30% higher in the bound state (Supplementary Table S1), likely indicating subtle differences in aggregation of solution versus AH-bound P[8] during heating.

## P[8] Antigen-AH Adjuvant-Thimerosal Interactions

**Figure 3:**
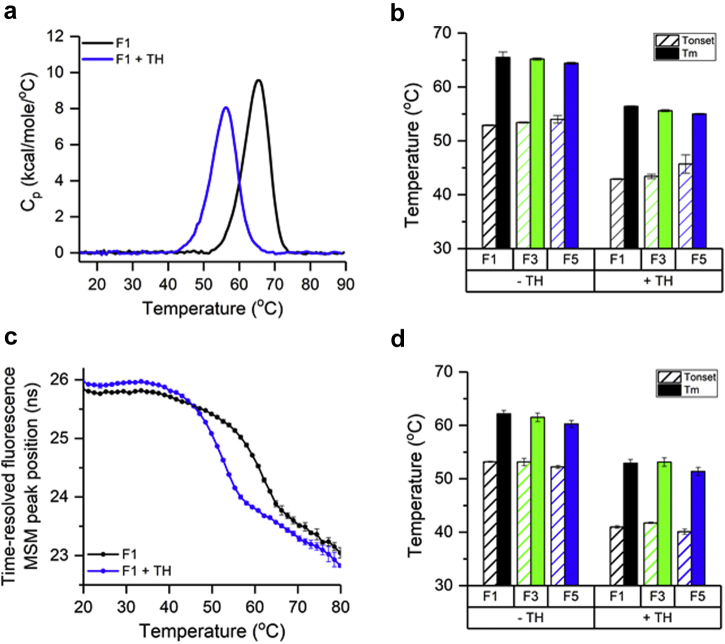
Effect of thimerosal on the conformational stability of Alhydrogel-bound P[8] antigen. (a) Representative DSC thermograms for P[8]-AH samples in formulation F1 with (blue) and without (black) thimerosal. (c) Time-resolved intrinsic fluorescence spectroscopy versus temperature of P[8]-AH samples in formulation F1 with (blue) and without (black) thimerosal. Thermal onset (T_onset_) and melting (T_m_) temperatures of (b) conformational stability (DSC) and (d) tertiary structure (intrinsic fluorescence) in formulations F1, F3, and F5. Error bars represent 1 SD from triplicate measurements. See Table 1 for composition of the different formulations. MSM, mean spectral mass.

The ZP of AH in 3 formulation buffers (F1, F3, F5) after pretreatment with 0.002%-0.05% (w/v) thimerosal did not change (values of +22 to +28 mV) indicating no interaction of AH with thimerosal (data not shown). P[8] was 100% bound to AH in the presence of 0.01% thimerosal at the clinical concentration (i.e., 60 μg antigen and 560 μg adjuvant per 0.5 mL dose) in each of the 3 formulations. Langmuir binding isotherms showed no significant changes in the adsorptive strength or capacity values of AH-P[8] interactions due to thimerosal addition (Table 1). However, 0.01% w/v thimerosal dramatically reduced the conformational stability of the bound P[8] as measured by DSC where the T_onset_ and T_m_ values decreased by ∼10°C and apparent enthalpy decreased by ∼20% (Fig. 3a and Supplementary Table S1 for formulation F1 and Fig. 3b for formulations F3, F5). Similar to DSC results, a reduction of ∼9°C in T_m_ values and ∼12°C in T_onset_ values was observed by thimerosal addition for aluminum-adsorbed P[8] as measured by time-resolved fluorescence (Figs. 3c and 3d).

### Storage Stability of Monovalent P[8]-AH Drug Product

The effect of thimerosal was evaluated during a 12-week stability study with monovalent P[8] antigen bound to AH at different storage temperatures (4°C, 25°C, 37°C) in different formulations (see Table 1 for composition). A schematic describing the vaccine preparation workflow and physicochemical attributes evaluated is provided in Supplementary Figure S1. Overall, 10 formulations were tested for their effects on antigen-adjuvant interactions as well as P[8] physicochemical stability and antigen-binding capacity during storage. As described below, although clear destabilizing effects of thimerosal were observed, the different buffering agents (phosphate or histidine) or surfactant concentration (0.025% or 0.006% w/v polysorbate-80) had no notable effects on the stability of aluminum adjuvant–bound P[8]. The results from all ten P[8] formulations are presented, nonetheless, to demonstrate the reproducibility of the destabilizing effects of temperature and thimerosal.

**Figure 4:**
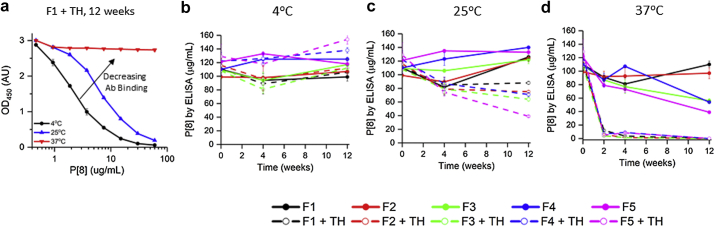
ELISA antibody binding for AH-bound P[8] antigen during storage stability studies at different temperatures in 10 different formulations. (a) Representative OD_450_ inhibition ELISA curves for P[8]-AH samples in F1 + TH formulation after 12 wk at different storage temperatures, (b-d) aluminum-adsorbed P[8] binding to antibody as determined from the inhibition ELISA assay over 12 wk of storage. Error bars represent 1 SD from triplicate vials. See Table 1 for composition of the different formulations. TH, 0.01% thimerosal.

Inhibition ELISA was used to measure the ability of adjuvant-bound P[8] to bind a specific antibody. Figure 4a shows representative data for P[8] antigen (formulation F1 + thimerosal) after 12 weeks storage at different temperatures. A clear shift in the OD450 curve is observed, which is indicative of reduced antibody binding. During 4°C storage, no notable differences were observed between P[8] formulations and time points with good antibody binding observed (Fig. 4b). At 25°C, the stability of P[8] formulated without thimerosal was similar to T0. In contrast, a decreasing trend in antibody binding was observed in P[8] formulations containing thimerosal (Fig. 4c). At 37°C, P[8] rapidly lost the ability to bind antibody in the presence of thimerosal with a slower but notable decreasing trend in P[8] formulations without thimerosal (Fig. 4d).

**Figure 5:**
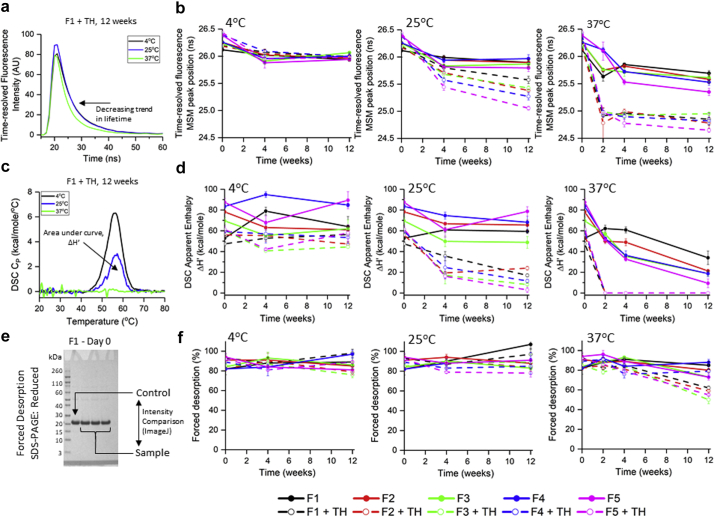
Physical stability profiles of AH-bound P[8] antigen during storage stability studies at different temperatures in 10 different formulations. Overall tertiary structure stability of P[8]-AH as measured by time-resolved fluorescence spectroscopy: (a) Representative fluorescence decay waveform data for P[8]-AH samples in formulation F1 + TH after 12 wk, (b) fluorescence lifetime MSM peak position or moment values for P[8]-AH samples stored in different formulations and at different temperatures. Conformational stability of P[8]-AH as measured by DSC: (c) Representative DSC thermograms for P[8]-AH sample in formulation F1 + TH, (d) apparent enthalpy of unfolding (ΔH’) values (or area under thermograms) over 12 wk of storage. Ability to desorb P[8] from AH as measured by SDS-PAGE: (e) Representative SDS-PAGE gel under reducing condition for P[8]-AH sample in formulation F1 at time zero, % desorption was calculated by comparing the intensity of desorbed P[8] band to control band by densitometry analysis using ImageJ, (f) % P[8] desorbed under forced desorption conditions. Error bars represent 1 SD from triplicate vials. See Table 1 for composition of the different formulations. TH, 0.01% w/v thimerosal.

To better understand thimerosal-induced loss of ELISA activity, the structural integrity of the P[8] antigen on the surface of AH was monitored by time-resolved fluorescence and DSC. As shown in Figure 5a, a faster decay of Trp fluorescence was observed in P[8]-AH samples stored at higher temperatures in the presence of thimerosal. Mean spectral mass peak position of the waveform (i.e., lifetime moment) was calculated and monitored over time. During storage at 4°C, 25°C, and 37°C (Fig. 5b), AH-bound P[8] undergoes structural alterations at elevated storage temperatures, which is accelerated by thimerosal. Overall, good stability was observed at 4°C in all formulations. For DSC analysis of the same samples, the total area of the thermogram (i.e., the apparent enthalpy of unfolding, ΔH’) reduced at T0 in the presence of thimerosal (Fig. 5c); however, no further notable losses were recorded during storage at 4°C (Fig. 5d). At 25°C and 37°C, P[8] formulations with thimerosal were much less stable with a decreasing trend in ΔH’ values over time (Fig. 5d). Overall, an excellent correlation was observed between P[8] stability trends comparing the ELISA antibody binding data with the biophysical measurements.

**Figure 6:**
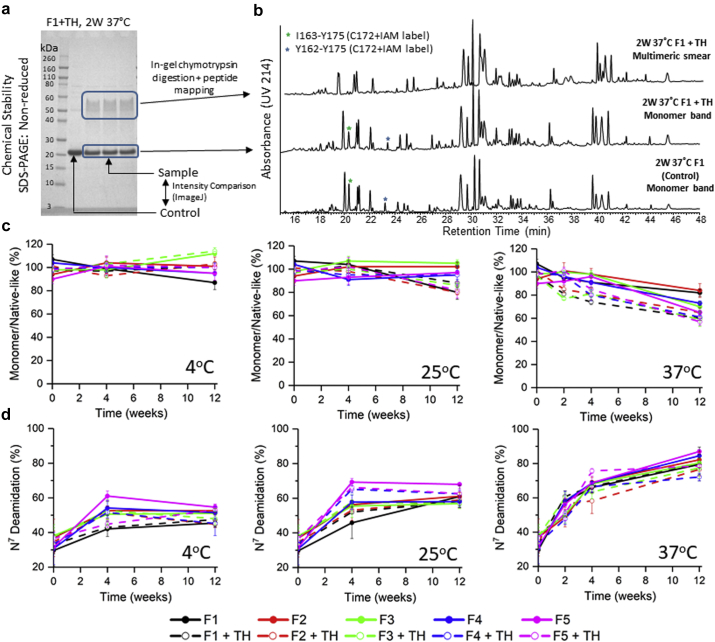
Nonnative disulfide formation at Cys^172^ and deamidation at Asn^7^ of the AH-bound P[8] antigen during 12 weeks of storage at different temperatures in 10 different formulations. (a) Representative SDS-PAGE gel under nonreducing condition for P[8]-AH sample in formulation of F1 + TH after storage for 2 wk at 37°C, % monomer/native-like species was calculated by comparing the intensity of desorbed P[8] monomer band to control band by densitometry analysis. (b) Representative peptide mapping chromatogram comparison of chymotrypsin-digested P[8] monomer and dimer bands from nonreduced SDS-PAGE gels for P[8]-AH sample in formulations F1 (control) and F1 + TH, 2-wk 37°C sample. The green and blue stars indicate UV_214_ peaks composed of 2 peptides containing IAM-labeled Cys^172^. (c) Percent monomer/native-like species from nonreduced SDS-PAGE analysis, and (d) relative deamidation of Asn^7^ over 12 wk of storage. See Table 1 for composition of the different formulations. Error bars represent 1 SD from triplicate vials. TH, 0.01% w/v thimerosal.

The interaction of P[8] antigen with the AH adjuvant over time was monitored by reduced SDS-PAGE with densitometry (Fig. 5e). Desorption of the AH-bound P[8] was carried out by adding SDS-PAGE sample buffer (with 0.2M phosphate and dithiothreitol added), heating the sample at 90°C, and subjecting the supernatant to SDS-PAGE. Essentially complete P[8] desorption from AH (∼80%-100%) was achieved throughout 12 weeks of incubation at 4°C or 25°C in all formulations (Fig. 5f and see Supplementary Fig. S3 for gels). At 37°C, however, a decreasing trend in % desorption was observed in thimerosal-containing formulations over time (Fig. 5f, dashed traces) indicating stronger binding of P[8] to AH over time. To better understand this observation, nonreduced SDS-PAGE analysis coupled to peptide mapping was also performed and the formation of dimeric P[8] species during storage of the aluminum-adsorbed antigen was observed (Figs. 6a and 6b). Overall, dimer formation was temperature dependent, and the rate/extent is increased in the presence of thimerosal (see Figs. 6c and Supplementary Fig. S3 for each gel). The dimeric species were not observed under reducing conditions indicating disulfide bond cross-linking between the P[8] monomers (see Supplementary Fig. S3), and this was confirmed by peptide mapping analysis (described below).

Nonreduced SDS-PAGE analysis coupled with in-gel chymotrypsin digestion and LC-MS peptide mapping identified 2 chemical changes in the AH-bound P[8] antigen during storage (nonnative disulfide bond formation at Cys^172^ and deamidation at Asn^7^). First, the P[8] monomeric versus dimeric bands were analyzed (Fig. 6b). At 37°C, 2 weeks, the chymotrypsin digested P[8] with thimerosal chromatograms were overall similar to the chromatograms of control P[8] without thimerosal except for peptides containing IAM-labeled Cys^172^ (observed in the control and the monomer band of the F1 + TH P[8] chromatograms, but absent in F1 + TH P[8] dimer band). These results show the observed P[8] dimer band on nonreduced SDS-PAGE was formed through a disulfide bond between single Cys^172^ of 2 P[8] monomers. Second, the levels of other PTMs in the P[8] monomer bands were evaluated at Asn^7^ and Met^99^ (Figs. 6d and Supplementary Fig. S4). Although some degradation of Asn^7^ and Met^99^ in P[8] occurred during SDS-PAGE and in-gel digestion analysis rather than during storage (data not shown), a stability trend for Asn^7^ deamidation was noted over 12 weeks of storage with highest levels in the samples stored at 37°C (∼80%), intermediate in the 25°C samples (∼60%), and lowest in 4°C samples (40%-50%).

### P[4] and P[6] Antigen-AH-Thimerosal Interactions

**Figure 7:**
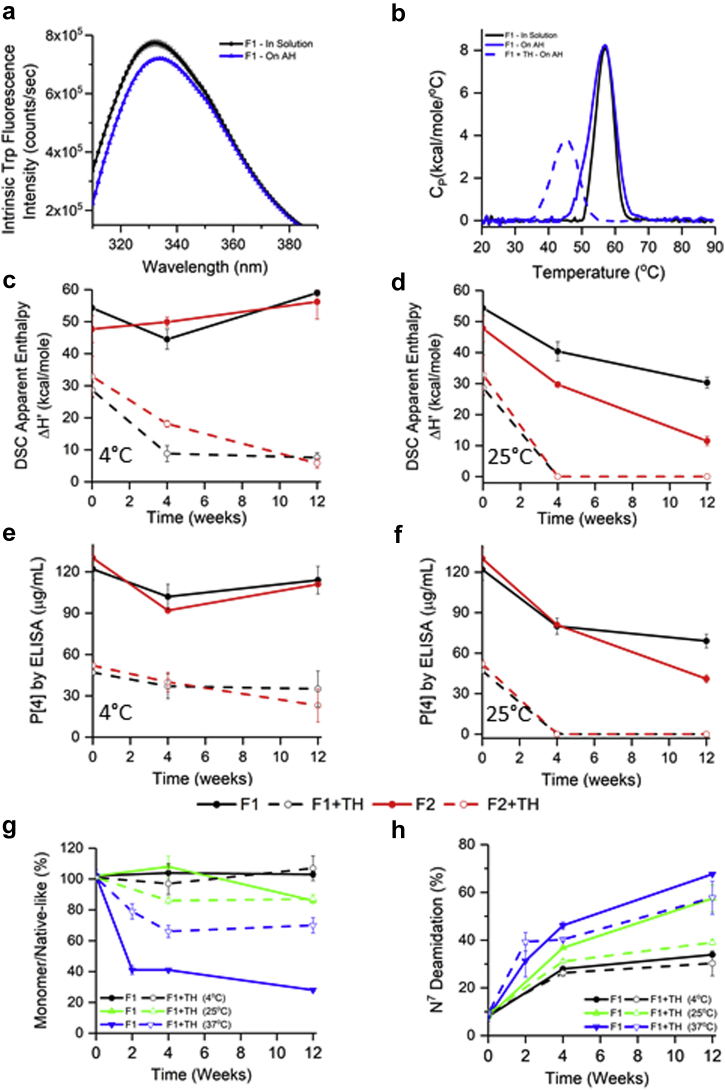
Effect of adjuvant (Alhydrogel, AH) and preservative (thimerosal) on physicochemical stability of P[4] antigen. (a) Tertiary structure integrity analysis using intrinsic tryptophan fluorescence emission spectra at 10°C in solution (black) and on AH (blue) in formulation F1 (see Supplementary Fig. S2C for data in formulations F3 and F5). (b) Conformational stability analysis with representative DSC thermograms of P[4] in solution (black) and bound to AH (blue) in F1 (see Supplementary Table S1 for T_onset_, T_m_, and ΔH’ values). Apparent enthalpy of unfolding (ΔH’) values of P[4]-AH from DSC at (c) 4°C and (d) 25°C storage temperatures over 12 wk (see Supplementary Fig. S5-A1 for 37°C data). Aluminum-adsorbed P[4] binding to antibody as determined from the inhibition ELISA assay over 12 wk of storage at (e) 4°C and (f) 25°C (see Supplementary Fig. S5-A2 for 37°C data). (g) Percent monomer/native-like P[4] species from nonreduced SDS-PAGE analysis (see Supplementary Fig. S6 for gels), and (h) relative deamidation of Asn^7^ from peptide mapping analysis, over 12 weeks of storage at different temperatures in F1 and F1 + TH (see Supplementary Fig. S8A for data in formulations F2 and F2 + TH). Error bars represent 1 SD from triplicate vials. See Table 1 for composition of the different formulations. TH, 0.01% w/v thimerosal.

**Figure 8:**
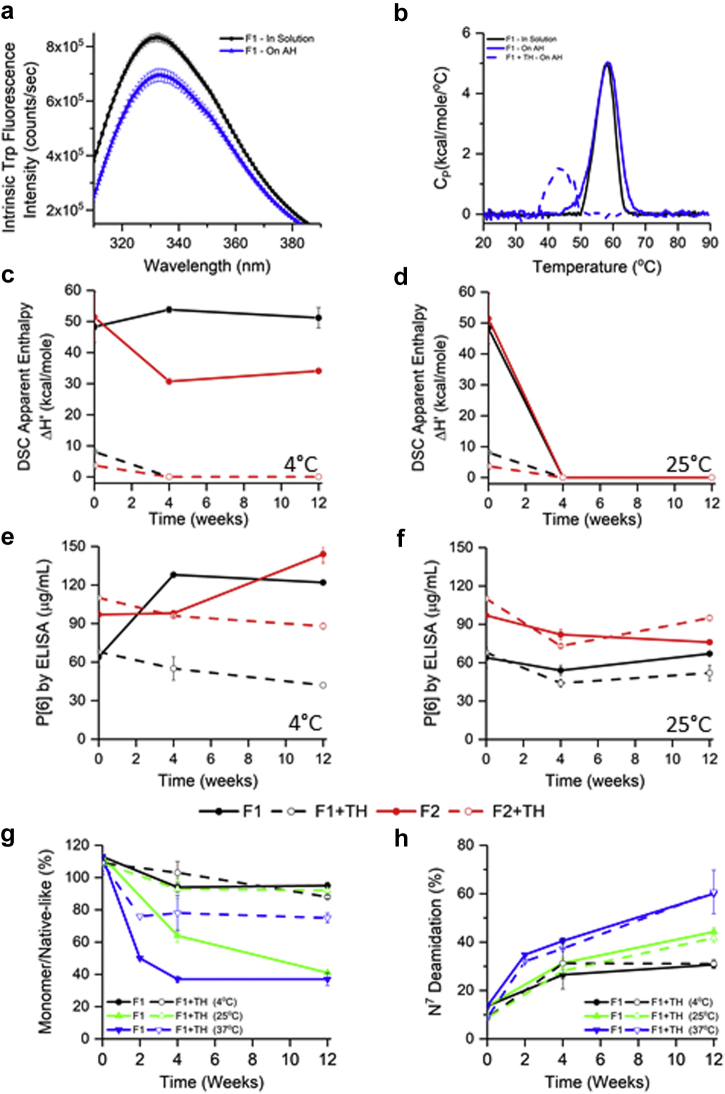
Effect of adjuvant (Alhydrogel, AH) and preservative (thimerosal) on physicochemical stability of P[6] antigen. (a) Tertiary structure integrity analysis using intrinsic tryptophan fluorescence emission spectra at 10°C in solution (black) and on AH (blue) in formulation F1 (see Supplementary Fig. S2B for data in formulations F3 and F5). (b) Conformational stability analysis with representative DSC thermograms of P[6] in solution (black) and bound to AH (blue) in F1 (see Supplementary Table S1 for T_onset_, T_m_, and ΔH’ values). Apparent enthalpy of unfolding (ΔH’) values of P[6]-AH from DSC at (c) 4°C and (d) 25°C storage temperatures over 12 wk (see Supplementary Fig. S5-B1 for 37°C data). Aluminum-adsorbed P[6] binding to antibody as determined from the inhibition ELISA assay over 12 wk of storage at (e) 4°C and (f) 25°C (see Supplementary Fig. S5-B2 for 37°C data). (g) Percent monomer/native-like P[6] species from nonreduced SDS-PAGE analysis (see Supplementary Fig. S7 for gels), and (h) relative deamidation of Asn^7^ from peptide mapping analysis, over 12 wk of storage at different temperatures in F1 and F1 + TH (see Supplementary Fig. S8B for data in formulations F2 and F2 + TH, and see Supplementary Fig. S9 for data showing deamidation of Asn^90^). See Table 1 for composition of the different formulations. TH, 0.01% w/v thimerosal.

Due to limited availability, only 3 formulations (F1, F3, and F5; see composition in Table 1) were selected to examine the stability of the monovalent AH-bound P[4] and P[6] antigens. Similar to P[8]-AH interaction results, the strength of adsorption was significantly higher in histidine formulations for P[4] and P[6] antigens compared with the phosphate-containing formulations (Table 1). The adsorptive capacity of AH was similar for P[4] in the 3 formulations, whereas some differences were observed for P[6] antigen (Table 1). Similar to P[8] results, P[4] and P[6] antigens also showed overall similar tertiary structures in solution or bound to AH by intrinsic fluorescence spectroscopy (Figs. 7a and 8a). From DSC analysis (Figs. 7b and 8b, and Supplementary Table S1), the T_m_ values of solution versus aluminum-bound P[4] and P[6] were comparable, whereas T_onset_ values were ∼5°C lower when bound to adjuvant. The addition of 0.01% thimerosal significantly reduced the conformational stability of P[4] and P[6] antigens.

During 12 weeks of storage (see Figure 7, Figure 8, and Supplementary Figs. S5-S9), the stability profiles of adjuvant-adsorbed monovalent P[4] and P[6] antigens were monitored by DSC, ELISA, and SDS-PAGE coupled to LC-MS peptide mapping. Similar to P[8] results mentioned previously, P[4] and P[6] showed good stability in the absence of thimerosal at 4°C up to 12 weeks as measured by conformational stability (Figs. 7c and 8c), ELISA antibody binding (Figs. 7e and 8e), and chemical stability (Figs. 7g and 8g). In the presence of thimerosal, notable destabilization of the P[4] and P[6] antigens was observed overall, albeit differences between antigens were noted depending on the method, temperature, and time point (see Figs. 7 and 8). For example with P[4]-AH samples, thimerosal and temperature induced loss of ELISA antibody binding showed an excellent correlation with loss of conformational stability data from DSC (Figs. 7c-7f, and Supplementary Fig. S5A). In contrast, no apparent effect of thimerosal was observed on P[6] antibody binding and the ELISA results did not correlate well with the DSC data. Polysorbate-containing formulations showed better antibody recognition compared with no polysorbate formulations, see Figures 8c-8f and Supplementary Fig. S5B. Finally, as observed with P[8] antigen, nonnative disulfide–linked dimeric species were observed for aluminum-adsorbed P[4] and P[6] antigens (see Supplementary Figs. S6 and S7 gels), and the abundance of monomer species decreased as a function of storage temperature as shown in Figure 7, Figure 8 and Supplementary Fig. S8. Overall, the P[6] antigen was most prone to this degradation pathway. Finally, increased levels of Asn^7^ deamidation in aluminum-adsorbed P[4] and P[6], and Asn^90^ deamidation in P[6] were recorded under accelerated storage temperatures (Figure 7, Figure 8 and Supplementary Figs. S8,S9).

### Comparison of Thimerosal versus 2-Phenoxyethanol on P[8]-AH Storage Stability

**Figure 9:**
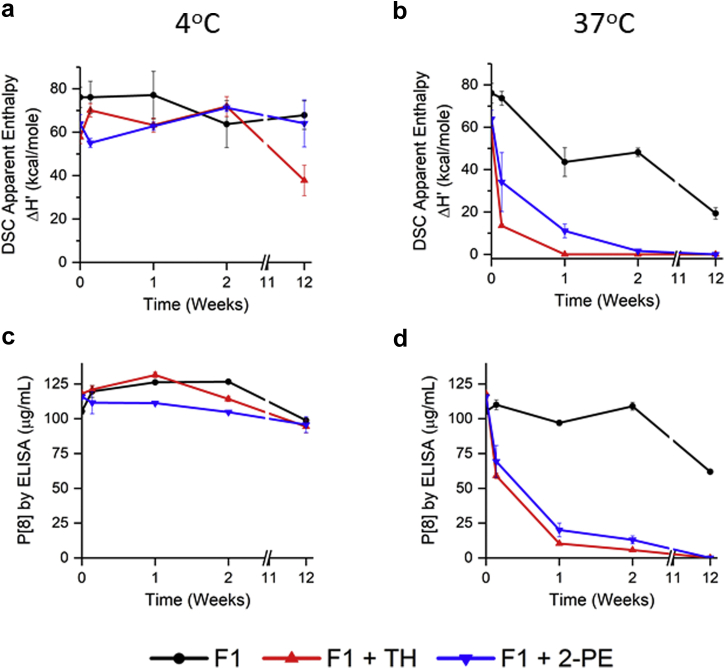
Destabilizing effect of commonly used concentrations of thimerosal (0.01% w/v) versus 2-PE (1.0% w/v) on conformational stability and antibody binding ability of AH-bound P[8] antigen. Stability study was over 12 weeks of storage at different temperatures in formulation F1. Apparent enthalpy of unfolding (ΔH’) from DSC at (a) 4°C and (b) 37°C storage temperatures. Aluminum-adsorbed P[8] binding to antibody as determined from the inhibition ELISA assay over 12 wks of storage at (c) 4°C and (d) 37°C. Error bars represent 1 SD from triplicate vials. See Table 1 for composition of F1. TH, 0.01% w/v thimerosal, 2-PE, 1.0% 2-phenoxyethanol.

Because thimerosal showed a detrimental effect on the storage stability of the 3 AH-bound monovalent NRRV antigens, the compatibility of another commonly used vaccine preservative (2-PE) was examined with AH-bound P[8]. During storage at 4°C (Figs. 9a and 9c), no notable changes were observed in P[8] stability as measured by conformational stability (DSC) or antibody binding (by ELISA), except P[8]-AH-thimerosal sample at 12 weeks which displayed some conformational instability by DSC. During storage at 37°C, a notable instability trend was observed for each of the P[8]-AH formulations, and the rate of degradation was fastest in the presence of thimerosal, followed by addition of 2-PE and then the no preservative control formulation (Figs. 9b, 9d, and S10). The DSC and ELISA results showed good agreement consistent with structural destabilization of AH-bound P[8] correlating with loss of antibody binding to the P[8] antigen.

## Discussion

In this work, the compatibility and stability of 3 recombinant fusion protein antigens (NRRV antigens P[4], P[6], P[8]; see Introduction) were assessed with 2 key components of a multidose subunit vaccine formulation, an aluminum adjuvant (AH) and 2 antimicrobial agents (thimerosal and 2-PE). The effect of formulation excipients/buffers on antigen-adjuvant interactions, antigen-preservative interactions, and storage stability of AH-bound NRRV antigens were also examined. The aim was to determine the feasibility of developing a multidose formulation of the NRRV candidate, and it was demonstrated that there are significant formulation and stability challenges for the NRRV antigens formulated with commonly used vaccine preservatives.

### NRRV Antigen-Aluminum Adjuvant (AH) Interactions

Adjuvants enhance vaccine immunogenicity and effectiveness by boosting components of the immune response (i.e., cellular, or humoral, or both) against the pathogen. Adjuvants can also facilitate dose sparing and reduce the number of shots.^22^ Aluminum-based adjuvants have been widely used in human vaccines for over 90 years and have a long record of safety and immunopotentiation.^23^ Antigen coprecipitation with formation of aluminum salts has in general been replaced with antigen adsorption to preformed aluminum adjuvants,^24^ Alhydrogel® (aluminum oxyhydroxide, AH) and Adju-Phos® (aluminum phosphate) are the 2 most commonly used aluminum adjuvants.^25^ NRRV antigens have isoelectric point of ∼6.0 and AH has point of zero charge ∼11.4. Thus, at physiological pH, AH is positively charged and the NRRV antigens are negatively charged, resulting in noncovalent electrostatic attractions. The current clinical NRRV formulation contains 0.5 mM phosphate ensuring complete antigen binding to AH; however, 0.5 mM phosphate provides a low buffering capacity.^7^^,^^10^ We demonstrated alternate buffering agents (Histidine, HEPES, Tris) could be used at higher concentration (10 mM) while maintaining 100% antigen binding (Fig. 1b). Lower binding strength was also observed for each NRRV antigen in phosphate buffer compared with histidine buffer (Table 1).

The binding of antigen to aluminum adjuvant has historically been considered important for generating immune responses, although that has more recently been shown to be antigen specific. Studies with poxvirus L1-protein antigen in our laboratories demonstrated AH binding is important for immunopotentiation,^26^ and others have reported similar observations with pneumococcal protein antigens.^27^ The WHO recommends greater than 80% of the diphtheria and tetanus toxoids to be adsorbed to aluminum adjuvants. Because many vaccine antigens require low microgram doses, unbound antigens can adsorb to various interfaces during fill-finish manufacturing^24^ so aluminum adjuvant binding can circumvent this and thus help reduce the cost of vaccine manufacturing. On the other hand, Hem et al. have shown that antigens formulated as unbound to aluminum adjuvant can elicit equal or better immune response compared with adjuvant-bound antigens.^28^, ^29^, ^30^ Hem et al. have shown that several model proteins that bind AH because of electrostatic attractions elute readily on contact with interstitial fluid,^31^ a potentially better parameter to predict immune responses.^32^ Finally, the strength of antigen adsorption to AH is another important parameter for immune responses. For example, inverse relationships have been reported for Hepatitis B surface antigen,^33^^,^^34^ HIV gp140 antigen,^35^ and anthrax recombinant protective antigen.^36^ Using a model protein, alpha casein, it has been shown that tighter aluminum adjuvant binding can diminish both T cell and B cell activation, by impairing antigen processing in dendritic cells and reducing antigen availability for B cell recognition, respectively.^37^ It will be of interest as part of future work to better characterize the effect of extent and strength of adsorption of NRRV antigens to AH in terms of immune response in animal models.

### Analytical Challenges to Evaluate NRRV Antigen Stability Bound to AH

Historically, animal-based immunogenicity tests have been used to assess the potency and stability of aluminum-adsorbed, inactivated/recombinant-based vaccines.^38^^,^^39^ There is great interest in replacing these *in vivo* assays with surrogate *in vitro* assays to monitor antigenicity and stability of vaccines. ELISA is a commonly used *in vitro* immunochemical method to measure the concentration, antigenicity, or conformational integrity of protein antigens, depending on the nature of the antibody used (e.g., neutralizing vs. nonneutralizing, linear vs. conformational epitopes, and so forth). In this work, a competitive ELISA format was used to measure the antibody binding ability of the aluminum-adsorbed NRRV antigens (McAdams et al., manuscript in preparation). The correlation of ELISA antibody binding results with NRRV antigen structural integrity is discussed below.

The use of physicochemical methods to monitor the structural integrity and stability of protein antigens in bound state to aluminum adjuvant is challenging because of low antigen doses, irreversibility of adsorption, and the turbid nature of the formulations. Examples of decreased,^40^^,^^41^ no effect,^42^^,^^43^ or even enhanced structural stability^44^^,^^45^ of various model proteins and recombinant vaccine antigens on adsorption to aluminum adjuvant have been reported. We have previously reported the development of a wide variety of analytical tools to monitor key structural attributes of the 3 NRRV antigens in solution and applied these methods to identify key degradation pathways.^19^^,^^20^ This work established that only a subset of these analytical methods could be applied to NRRV antigens bound to AH, and only a subset of possible degradation pathways are observed in the bound state. The chemical stability of AH-bound NRRV antigens was monitored by SDS-PAGE coupled with LC-MS peptide mapping. We noted that formation of nonnative, intermolecular disulfide bond at Cys (at position 173–P[4], P[6] and 172–P[8]) and deamidation of Asn^7^ are the 2 major chemical alterations for the NRRV antigens when bound to AH. The degree of Asn deamidation in protein antigens could potentially be enhanced on the surface of AH because of higher pH of the microenvironment^46^, ^47^, ^48^ leading to Asn deamidation–induced loss of potency, for example, as observed with a recombinant protective antigen vaccine. Estey et al.^49^ have reported for 3 recombinant botulinum protein antigens elevated levels of oxidation and deamidation in the adjuvant bound versus solution state.

The higher-order structure of the 3 NRRV antigens bound to AH was evaluated by a combination of fluorescence spectroscopy and DSC. Although NRRV antigens unfold and aggregate on heating in solution, when adsorbed to the surface of AH, the NRRV antigens undergo structural alterations but do not aggregate (due to spatial distances between the surface-bound protein molecules and inability to colloidally associate). Interestingly, this resulted in the apparent enthalpy of unfolding values for the NRRV antigens being higher on AH with a broader transition and a loss in the apparent enthalpy of unfolding (reduced area of thermograms) during storage (see next section). The AH-bound P[6] was most unstable of the 3 NRRV antigens, and this result aligns with the previous in solution characterization work with the 3 NRRV antigens.^19^ The addition of thimerosal showed a clear destabilizing effect (8°C-14°C) on the conformational stability of each NRRV antigen as measured by DSC and fluorescence spectroscopy. To better understand the effect of preservatives on the stability of aluminum-adsorbed NRRV antigens, a stability study was set up as described below.

### Formulation and Stability Challenges With NRRV Antigens in the Presence of Preservatives

These studies are a first step in the longer term goal of NRRV development to combine it with pentavalent childhood combination vaccines (e.g., diphtheria, tetanus, and pertussis, HepB, Hib) to enhance RV vaccine coverage and further lower costs. The most commonly used pediatric combination vaccine in the developing world contains both wcP and thimerosal. Thimerosal is used both as a preservative in the formulation and an inactivating agent during wcP production.^16^ A 12-week accelerated and real-time stability study was performed with each monovalent NRRV antigen bound to AH as evaluated by ELISA and physicochemical assays (as mentioned previously). Overall, no notable effect of buffer type or surfactant concentration was observed on NRRV antigen stability while thimerosal (and 2-PE) demonstrated a temperature-dependent detrimental effect on the stability of each aluminum-adsorbed NRRV antigen.

One measure of the prominent destabilizing effect of thimerosal on the conformational stability of AH-adsorbed NRRV antigens was measured by DSC with a dramatic reduction in ΔH’ of unfolding values during storage at elevated temperatures. This result implies some population of the AH-bound protein remains in native state, whereas others lose structural integrity, thus forming conformationally heterogeneous populations over time. Similar loss in DSC signal or ΔH’ of unfolding has been reported for recombinant botulinum neurotoxin adsorbed to AH after storage at 30°C for 9 weeks.^50^ Furthermore, protein structural changes on the surface of aluminum could lead to stronger adsorption over time, which in fact was observed for the thimerosal-containing P[8]-AH samples stored at 37°C for 12 weeks. For AH-bound P[8] and P[4] antigens, an overall good correlation was observed between the ELISA antibody binding and DSC results, suggesting the conformational nature of the antibody-binding epitopes. In the case of aluminum-adsorbed P[6] antigen, such a correlation is less convincing and additional work is in progress to better understand the conformational versus linear nature of this antibody-binding epitope (McAdams et al., manuscript in preparation).

Chemical stability assessment by SDS-PAGE and LC-MS peptide mapping analyses revealed the susceptibility of each AH-bound NRRV antigen to nonnative intermolecular disulfide formation via a single Cys residue. The extent/rate of this degradation was temperature dependent (37°C > 25°C > 4°C), and P[6] was most susceptible. Addition of thimerosal clearly exacerbated disulfide formation for the P[8] antigen, whereas for P[4] and P[6], disulfide formation was unaffected or perhaps somewhat prevented by addition of thimerosal.

Thimerosal is an organometallic compound which degrades in aqueous solution into thiosalicylic acid and ethyl mercury.^51^ Ethyl mercury can bind to free Cys in proteins, which leads to ethyl mercury adduct formation.^52^^,^^53^ Ethyl mercury adducts were observed for NRRV antigens in the presence of thimerosal by intact mass analysis (manuscript in preparation). We hypothesize that the interaction between free Cys and ethyl mercury leads to structural/conformational perturbation of the surrounding domains in the protein which ultimately leads to protein structural alterations. The degree of adduct formation could also depend on the accessibility of the free Cys within each NRRV antigen’s 3-dimensional structure, which in turn could increase at elevated storage temperatures because of increase in protein dynamics. In contrast, no effect of thimerosal was seen on the extent of Asn^7^ deamidation in each antigen which could be due to the spatial distance of this residue from the thimerosal-affected Cys-containing domain of the protein. As expected, the extent of Asn deamidation increased at higher storage temperatures in each antigen.

We also tested the compatibility of a second commonly used vaccine preservative 2-PE with the P[8] antigen, and similar destabilizing trends were observed as seen with thimerosal as measured by ELISA and DSC. This result implies the destabilization of the NRRV antigens by preservatives is more complex mechanistically than direct interaction with the free Cys residue of the NRRV antigen (because 2-PE lacks the sulfhydryl chemistry described previously for thimerosal). 2-PE is also known to destabilize a model protein cytochrome *c* and therapeutic proteins including an IgG1 mAb and interferon α-2a by causing structural perturbations and aggregation.^54^, ^55^, ^56^

### Ongoing and Future Work

Ongoing work in our laboratories is focused on elucidating the molecular mechanism of thimerosal-induced destabilization of the NRRV antigens (manuscript in preparation), evaluating other preservatives, and reengineering the NRRV antigens through point mutations (e.g., replacement of the single Cys residue) to potentially produce NRRV antigens that are more compatible with preservatives. The overall goal of these various approaches is to facilitate development of multidose formulations of NRRV, ideally as part of currently available pediatric combination vaccines, to produce a lower cost RV vaccine with wider vaccine coverage for use in the developing world.
