# Supplementary material for: Effect of Aluminum Adjuvant and Preservatives on Structural Integrity and Physicochemical Stability Profiles of Three Recombinant Subunit Rotavirus Vaccine Antigens
Source: J Pharm Sci. 2020 Jan;109(1):476–87. doi: 10.1016/j.xphs.2019.10.004 (PMC6941222; doi:10.1016/j.xphs.2019.10.004)
Supplement: Supplemental Information [file mmc1.docx]

**Supplemental Information**

**Effect of aluminum adjuvant and preservatives on structural integrity and physicochemical stability profiles of three recombinant subunit rotavirus vaccine antigens**

Sanjeev Agarwal^a^, John M. Hickey^a^, David McAdams^b^, Jessica A. White^b^, Robert Sitrin^c^, Lakshmi Khandke^c^, Stanley Cryz^c^, Sangeeta B. Joshi^a^, and David B. Volkin^a,*^

^a^ Department of Pharmaceutical Chemistry, Vaccine Analytics and Formulation Center, University of Kansas, 2030 Becker Drive, Lawrence, Kansas 66047, USA

^b,c^ The Center for Vaccine Innovation and Access, PATH

^b^ 2201 Westlake Avenue, Suite 200, Seattle, WA 98121 USA

^c^ 455 Massachusetts Ave NW Suite 1000, Washington, DC 20001, USA

**Current address:** Sanjeev Agarwal: Amgen, Thousand Oaks, CA, 91320, USA

*** Correspondence to:** David B. Volkin: 2030 Becker Drive, Lawrence, KS 66047, Email: volkin@ku.edu; Phone: 785-864-6262; Fax: 785-864-5736

**Supplemental Methods**

**Measuring Zeta Potential**

Zeta potential of AH was measured in different buffers using ZetaPALS Zeta Potential Analyzer (Brookhaven Instruments Corporation, NY). AH samples were prepared at 1.5 mg/mL of aluminum at varying concentrations of the buffering agent (0, 0.5, 1.0, 2.5, 5.0, 10, 20, 50, 100 mM), 150 mM NaCl and 0.025% PS-80. The different buffering agents tested were sodium phosphate, HEPES and Tris at pH 7.2, and Histidine at pH 6.5, 6.8 and 7.2. Sample were incubated overnight at RT and 1.5 mL of the diluted AH sample was used for the measurement in a disposable plastic cuvette and appropriate electrode. Each buffer condition was measured in duplicate and for each measurement 10 runs were collected with 10 cycles per run. All measurements were conducted at 25˚C and parameters such as viscosity, refractive index and dielectric constant were chosen for water. Electrophoretic mobility was used by the instrument software to calculate zeta potential using Smoluchowski approximation. Finally, the average and 1SD of the 20 runs were reported for each buffer condition as the measured Zeta potential value of AH.

For the preservative containing samples, AH samples were prepared at 1.5 mg/mL of aluminum in formulations F1, F3 and F5 (see Table 1 in main text for formulation composition) with increasing amounts of thimerosal (0, 0.002, 0.005, 0.01, 0.02, 0.05% w/v). Samples were incubated overnight at room temperature and analyzed the following day is similar way as described above.

**Antigen-Adjuvant Binding Study**

Binding of NRRV antigens to AH was studied at their clinical concentration (i.e., 60 µg of antigen and 560 µg aluminum as AH per 0.5 mL) in different buffering agents in the presence of 150 mM NaCl and 0.025% PS-80. Each protein sample at 2X concentration was mixed 1:1 with 2.24 mg/mL AH (in 150 mM NaCl) to achieve the final desired concentration of protein, aluminum and buffer in the final formulation. After mixing, the antigen adsorbed samples were incubated overnight at 4˚C and then centrifuged at 5,000 X g for 10 min. The amount of unbound protein in the supernatant was measured by UV-Visual spectroscopy using an Agilent 8453 UV-Visible Spectrophotometer (Palo Alto, CA).

**Langmuir Binding Isotherms**

Appropriate volume of antigen stock, PS-80 stock and buffer were mixed in an Eppendorf tube to a final volume of 250 µL containing 0, 20, 50, 75, 100, 150, or 200 µg of antigen and 0.05% PS-80. These protein solutions were mixed 1:1 with AH solution (0.2 mg/mL aluminum in saline) to get the desired amount of antigen (i.e. 0, 10, 25, 37, 50, 75, or 100 µg), buffering agent, and PS-80. Samples were incubated overnight at 4˚C and then centrifuged at 5,000 X g for 10 min to pellet the adjuvant and bound antigen fraction. UV-visible spectroscopy was used to measure the concentration of free/unbound antigen in the supernatant.

Once the concentration of free antigen (C_e_) had been determined for each total added amount of antigen (C_o_) in the antigen-adjuvant mixture, the amount of bound antigen could be calculated (C_o_ - C_e_). Then the amount of bound P[8] per mg of aluminum (Q_e_) can be calculated by dividing C_o_ - C_e_ by the amount of aluminum used (i.e., 50µg). Finally Langmuir isotherm was generated by plotting Q_e_ vs C_e_ and linear form of the isotherm can be obtained by plotting C_e_/Q_e_ vs C_e_. Linear form of the Langmuir isotherm was further analyzed to obtain the adsorptive capacity (Q_m_) from the slope and adsorptive/Langmuir coefficient (K_L_) from the y intercept of the fitted straight line. For the studies in the presence of thimerosal, 0.01% w/v thimerosal was added after binding the antigen to adjuvant, and samples were incubated overnight at 4˚C prior to the generation of isotherms. Micro-BCA assay (ThermoFisher Scientific, MA) was used to measure the concentration of free/unbound antigen due to interference from thimerosal absorbance in the UV region.

**Steady State Intrinsic Tryptophan Fluorescence**

The intrinsic tryptophan fluorescence of NRRV antigens was measured in solution and on AH using a Photon Technology International (PTI) spectrofluorometer (Lawrenceville, NJ) equipped with a turreted four-position Peltier-controlled cell holder and a xenon lamp. For in solution samples, spectra could be collected in a conventional way by pouring 0.7 mL of sample in 1 X 1 cm path length quartz cuvette. However, for adjuvant bound samples, 0.2 mg/mL of antigen stock was mixed 1:1 with 4 mg/mL of aluminum. Two milliliter of this sample was added to the 1 X 1 cm quartz cuvette and then the samples were allowed to settle overnight at 4˚C (or centrifuged at 100 X g for 1 min to pellet the adjuvant bound protein). Following day, the fluorescence emission spectra were recorded as a function of temperature (10 – 90˚C) as described previously ^1^. For studies in the presence of preservative, 0.01% thimerosal was added after binding the antigen to adjuvant and samples were incubated overnight at 4˚C.

**Differential Scanning Calorimetry (DSC)**

DSC was performed for the NRRV antigens at 0.12 mg/mL in solution and on AH using an Auto-VP capillary differential scanning calorimeter (MicroCal/GE Health Sciences, Pittsburgh, PA) as described before ^1^.

**Time-resolved Intrinsic Tryptophan Fluorescence**

Intrinsic tryptophan fluorescence lifetime for NRRV antigens bound to AH ± 0.01% w/v thimerosal was obtained using a fluorescence plate-reader (Fluorescence Innovations, Minneapolis, MN) equipped with a tunable pulsed dye laser, a high-speed digitizer and a temperature controlled 384-well sample holder (Torrey Pines Scientific, Carlsbad, CA). This fluorometer has two detectors: a charge coupled device (CCD) spectrometer and a photomultiplier tube (PMT), permitting recording of steady-state fluorescence and time-resolved intrinsic fluorescence, respectively. The emission signal was collected at 180° (front face geometry) and then sent through a 300 nm long pass filter (Thorlabs Inc., Newton, NJ). For time-resolved fluorescence recording, the signal was further passed through a 360 nm (± 23 nm) band-pass filter before reaching the PMT. Samples were loaded (10 µL) into a 364 well plate (Hard-Shell 384-well PCR plates) and 2 µL silicon oil (ThermoFisher Scientific) was added on top to avoid sample evaporation. Excitation wavelength of 295 nm was used and temperature ramp was set from 10-90˚C with step size of 1.25˚C and 2 min equilibration at each temperature. Time-resolved intrinsic fluorescence was performed by recording the fluorescence lifetime decay waveforms within a time scale of 100 ns. Life-time moment (ns) was obtained from the fluorescence lifetime decay waveform which is defined as the center of the waveform which vertically divides the peak area in two halves. Moment is similar to mean spectral mass in steady state intrinsic Trp fluorescence experiments. The lifetime moment represents the intensity-averaged lifetime of the waveform plot and its mathematical definition has been previously described ^2^.

**SDS-PAGE Analysis + LC-MS Peptide Mapping, of desorbed protein**

Antigen-adjuvant sample (500 µL, 60 µg antigen + 560 µg aluminum) was centrifuged at 4,000 X g for 5 min to pellet the adsorbed antigen and adjuvant. Then 460 µL the supernatant was removed and the pellet was re-suspended in a mixture of 0.2M sodium phosphate + LDS buffer (Life Technologies) + 20 mM Iodoacetamide (Thermo Scientific), and incubated in dark for 15 min at RT. Then the samples were heated at 90˚C for 10 min, followed by centrifugation at 4,000 X g for 5 min. Supernatant was recovered and divided in two parts to prepare non-reduced and reduced samples for SDS-PAGE analysis. For reduced samples, supernatant was mixed with 10 mM DTT (Thermo Scientific) and, for non-reduced samples, equal volume of ultra-pure water was added and then the samples were incubated at 37˚C for 15 min. Finally, the reduced/non-reduced samples were separated by SDS-PAGE gel electrophoresis using NuPAGE 4-12% Bis-Tris (Life Technologies) gels and a MES running buffer (Life Technologies). A theoretically equivalent amount of protein was also loaded on the gel as in-solution control (i.e., protein that was never exposed to adjuvant). The purpose of running in-solution control was to quantify % desorption under forced desorption condition of phosphate + LDS sample buffer + boiling at 90°C for 10 min by comparing the band intensities between in-solution control and desorbed samples by ImageJ (NIH, US) analysis. Gels were run for first 10 min at 120V followed by 50 min at 150V. Protein bands were visualized by staining with coomassie blue R250 (Teknova, Hollister, CA) for 1 hr and destained with a mixture of 40% methanol, 10% acetic acid, and 50% ultrapure water. Gels were digitized using an Alphaimager (Protein Simple, Santa Clara, CA) gel imaging system.

For LC-MS peptide mapping, each monomer band and selected dimeric bands were excised from the SDS-PAGE gel and cut into small pieces. The Coomassie stain in the gel pieces was removed through two 45 min washes at 37˚C with 0.2 M ammonium bicarbonate pH 7.5 + 50% acetonitrile. The gel pieces were then dehydrated using a SpeedVac (Eppendorf, Hamburg, Germany) for ~30 min at 30˚C and then rehydrated with 50 mM ammonium bicarbonate pH 7.5. Chymotrypsin (3.5 µg, Promega, Madison, WI) was added and the samples were incubated overnight at 37˚C. The following day the solution in each sample was removed and 0.05% trifluoroacetic acid was added to inactivate proteolysis. The samples were then subjected to LC-MS peptide mapping.

The peptides from each digested protein solution were separated by a liquid chromatography system (Thermo Scientific, Waltham, MA) prior to analysis. Peptides were injected onto a C18 column (1.7µm, 2.1 x 150 mm, Waters) and a 55 min 5-50% B gradient (A: H2O and 0.04% trifluoroacetic acid; B: ACN and 0.04% trifluoroacetic acid; 200 μl/min flow rate) for separation. MS was performed using a LTQ-XL ion trap (Thermo Scientific) and the Xcalibur 2.0 software (Thermo Scientific). The instrument was also tuned using a standard calibration peptide (Angiotensin II, Sigma) for maximal sensitivity before running any experiments. The mass spectra were acquired in the LTQ over a mass range of m/z 350-1900. The ion selection threshold was 10,000 counts and the dynamic exclusion duration was 8 sec.

Raw experimental files were initially evaluated manually to determine if the ion counts and fragmentation of each peptide were sufficient for further analysis. The raw data files were then processed using PepFinder 2.0 software (Thermo Scientific). The database used for this experiment consisted of the [P8] and chymotrypsin primary sequences. Potential post-translational modifications (Asn deamidation and Met oxidation) were included during the analysis. Peptide assignments of MS/MS spectra were validated using a confidence score of ≥ 95%.

**Inhibition ELISA**

The details of the inhibition ELISA assay used in this work, including the antibodies used and the nature of their interaction with NRRV antigens, is described elsewhere (McAdams *et al*., manuscript in preparation). Briefly, the AH bound NRRV antigen samples were first incubated with a blocking buffer, then serial dilutions were made and incubated with a fixed amount of NRRV P[x] antigen specific antibody (primary antibody) overnight. Samples were centrifuged and the supernatant containing free antibody was transferred to a 96 well plate coated with the NRRV P[x] antigen standard. The plate was incubated at room temperature for two hours and then the amount of primary antibody bound on the plate was determined with a horseradish peroxidase labeled secondary antibody using a tetramethylbenzidine substrate. OD450 values were recorded using a SpectraMax® plate reader (Molecular Devices). The NRRV P[x] antigen levels in the test samples were calculated by comparing results to OD450 values of a NRRV P[x] antigen reference standard using multi-parameter fitting of the standard curve.

**Accelerated/Real Time Storage Stability Study**

Monovalent NRRV vaccines (mock, lab scale) were prepared in five formulations for P[8] with and without 0.01% thimerosal as illustrated in the schematic shown in Sup. Figure S1. Total ten formulations (F1 – F5, ± 0.01% w/v thimerosal) were tested with P8 antigen bound to AH and details of formulation composition is provided in Table 1 in main text. Due to limited availability of P[4] and P[6], four formulations (F1, F2, ± 0.01% w/v thimerosal) were tested with these antigens. Adsorption was achieved by adding the 2X antigen stock (sterile filtered through a 0.22 µm filter) to the 2X AH stock with gentle mixing and final concentrations of protein and aluminum were 0.12 mg/mL and 1.12 mg/mL, respectively. Two milliliter aliquots of vaccine were dispensed into 3 mL Fiolax Clear glass vials (West Pharmaceutical Services, PA) and rubber stoppered (NovaPure®, West Pharmaceutical Services, PA). Vaccine vials were sealed with flip off caps and were incubated at 4ºC and 25ºC storage temperatures for 4 and 12 weeks, and at 37ºC for 2, 4, and 12 weeks. At each time point, samples were pulled from the incubator and assayed for different physicochemical characteristics. As shown in the schematic in Sup. Figure S1, HOS integrity, conformational stability and antibody binding were studied with antigen in the bound state, whereas, chemical stability, monomer quantitation and desorption quantitation analysis were carried out after forced desorption of the antigen. For the storage stability assessment of monovalent P[8] vaccine in the presence of 1.0% w/v 2-Phenoxyethanol, vaccine vials were stored at 4ºC and 37ºC for 1, 7, 14 and 84 days.

**References for Supplemental Information**

1. Agarwal S, Hickey JM, Sahni N, Toth RT, Robertson GA, Sitrin R, Cryz S, Joshi SB, Volkin DB 2019. Recombinant subunit rotavirus trivalent vaccine candidate: physicochemical comparisons and stability evaluations of three protein antigens.

2. Wei Y, Larson NR, Angalakurthi SK, Russell Middaugh C 2018. Improved Fluorescence Methods for High-Throughput Protein Formulation Screening. SLAS Technol 23(6):516-528.

**Supplementary Table S1.** Summary of onset temperature (T_onset_), melting temperature (T_m_) and apparent enthalpy of unfolding (ΔH’) values from conformational stability analysis by DSC for the three NRRV antigens in solution and when bound to Alhydrogel adjuvant (AH) in the presence and absence of 0.01% w/v thimerosal. Data shown are for F1 formulation, 0.5 mM Sodium Phosphate, 0.15 M NaCl, pH 7.2. Error bars represent 1 SD from triplicate measurements.

|  |  | In Solution | | On Adjuvant | |
| --- | --- | --- | --- | --- | --- |
|  |  | No Preservative | 0.01% Thimerosal | No Preservative | 0.01% Thimerosal |
| T_onset_  (°C) | P[4] | 49.8 ± 0.3 | 39.2 ± 1.1 | 44.6 ± 1.1 | 35.8 ± 1.1 |
|  | P[6] | 49.9 ± 0.1 | 38.3 ± 0.3 | 45.4 ± 0.8 | 36.8 ± 0.1 |
|  | P[8] | 54.2 ± 0.4 | 46.5 ± 0.1 | 52.9 ± 0.1 | 42.9 ± 0.1 |
| T_m_  (°C) | P[4] | 57.0 ± 0.0 | 47.8 ± 0.1 | 56.9 ± 0.1 | 45.1 ± 0.1 |
|  | P[6] | 57.9 ± 0.1 | 47.7 ± 0.0 | 58.3 ± 0.3 | 43.9 ± 0.2 |
|  | P[8] | 63.9 ± 0.1 | 56.2 ± 0.1 | 65.5 ± 0.1 | 56.4 ± 0.1 |
| ΔH’  (kcal/mole) | P[4] | 51.5 ± 0.6 | 33.8 ± 1.7 | 75.0 ± 1.3 | 29.6 ± 9.4 |
|  | P[6] | 36.6 ± 2.2 | 18.9 ± 0.6 | 45.5 ± 6.9 | 10.4 ± 0.8 |
|  | P[8] | 66.4 ± 0.4 | 51.7 ± 3.5 | 86.0 ± 0.6 | 71.0 ± 5.5 |

**Supplementary Figure S1.** Schematic description of the mock, lab scale NRRV vaccine sample preparation work-flow in ten candidate formulations with and without 0.01% w/v thimerosal for 12 week storage stability study. Different analytical methods were used for structural integrity and physicochemical stability assessments with antigen either bound to aluminum adjuvant or after forced desorption. Refer to Table 1 in main text for the composition of each formulation.


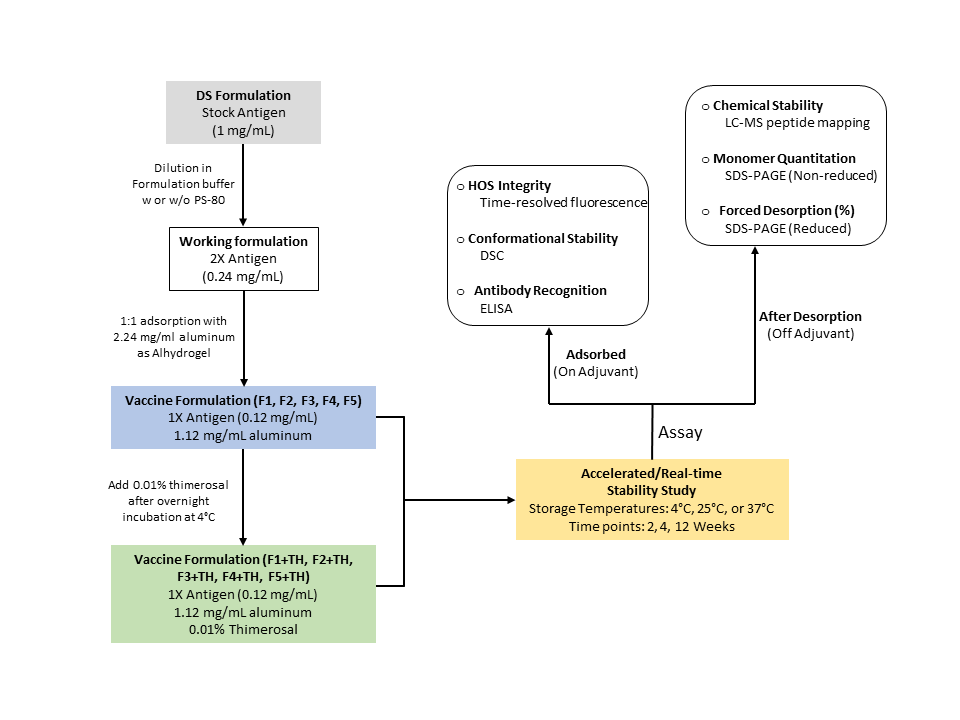


**Supplementary Figure S2.** Tertiary structure integrity and stability analyses of NRRV antigens bound to aluminum adjuvant Alhydrogel, AH (○) compared to solution controls (●) in three different formulations. Intrinsic Trp fluorescence (A1, B1, C1) emission spectra at 10°C, and (A2, B2, C2) MSM peak intensity, and (A2, B2, C2) MSM peak position vs. temperature for the three NRRV antigens. Error bars represent 1 SD from triplicate measurements. Refer to Table 1 in main text for the composition of each formulation.


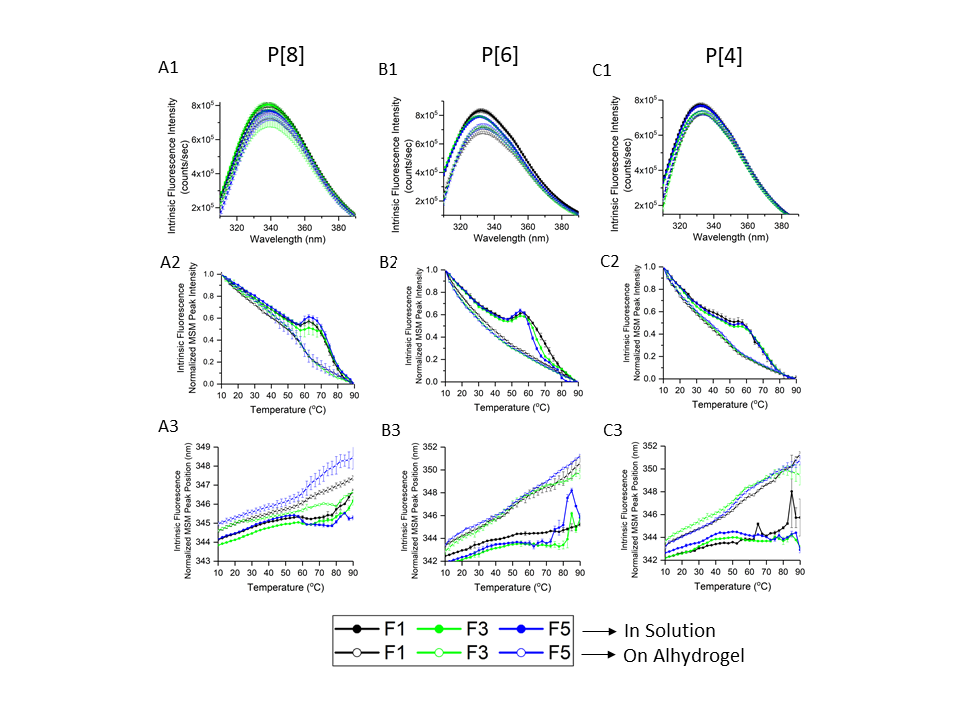


**Supplementary Figure S3.** SDS-PAGE analysis of P[8] antigen under non-reducing and reducing conditions after forced desorption from Alhydrogel adjuvant at different time points during 12 week storage at different temperatures. TH – 0.01% w/v thimerosal. Refer to Table 1 in main text for the composition of each formulation.


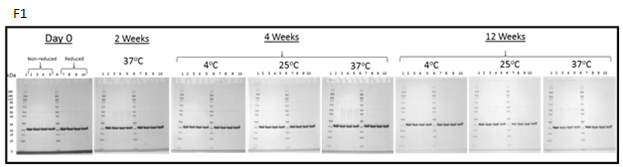


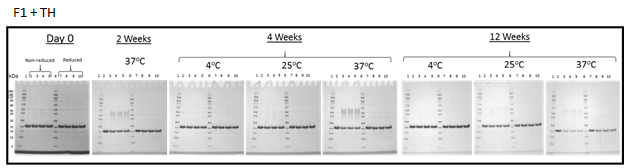


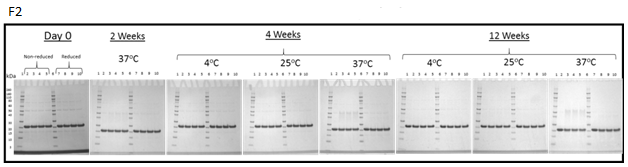


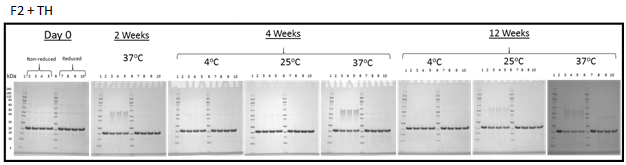


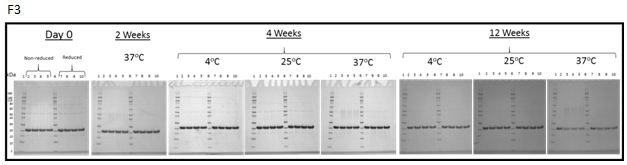


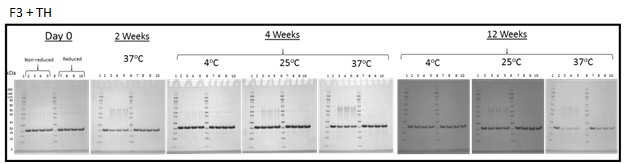


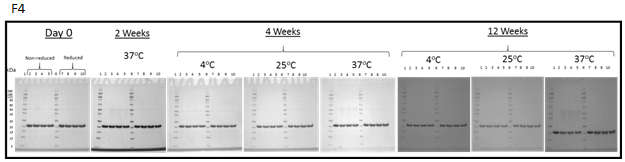


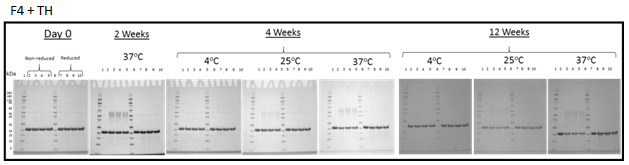


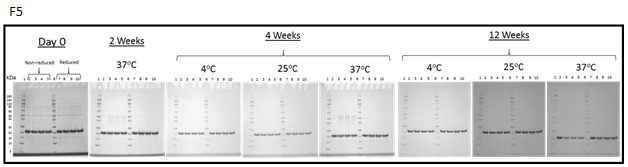


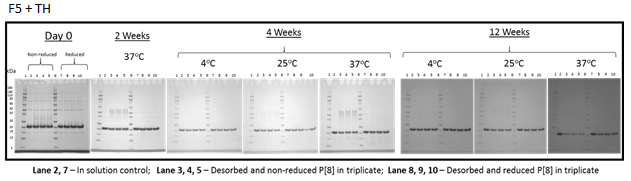


**Supplementary Figure S4.** Oxidation propensity of Met^99^ of the P[8] antigen bound to Alhydrogel adjuvant during 12 weeks of storage at different temperatures in ten different formulations. Relative oxidation values from LC-MS peptide mapping analysis. Error bars represent 1 SD from triplicate vials. Refer to Table 1 in main text for the composition of each formulation. TH – 0.01% w/v thimerosal.


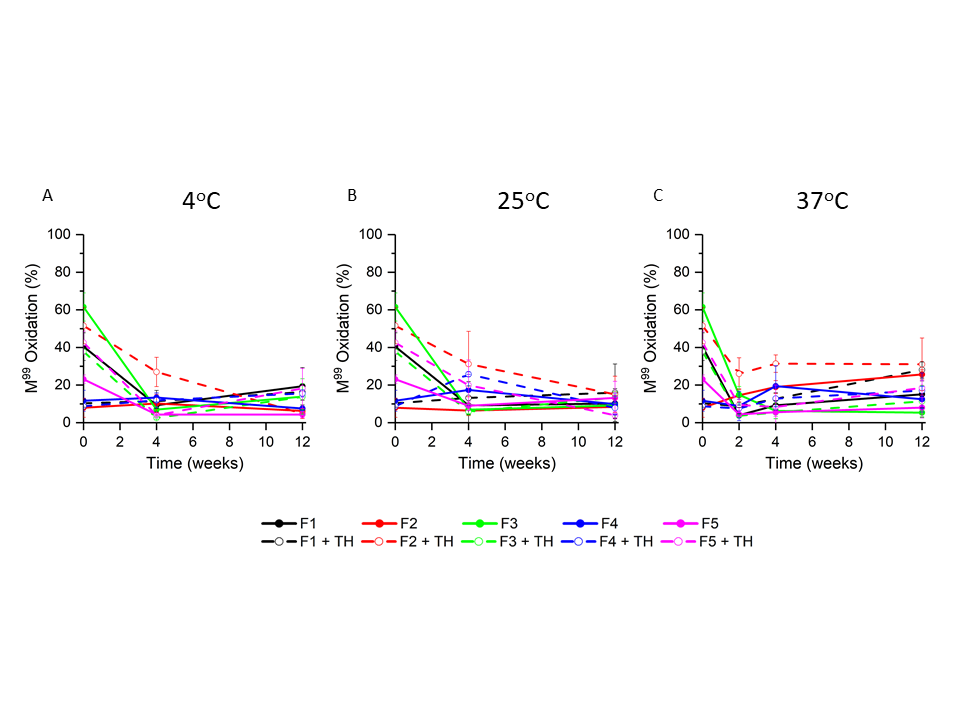


**Supplementary Figure S5.** Conformational stability and antibody binding of monovalent P[4] and P[6] antigens bound to Alhydrogel adjuvant during 12 weeks of storage at 37°C. (A1, B1) Apparent enthalpy of unfolding (ΔH’) from DSC, and (A2, B2) antigen binding to antibody as determined from the inhibition ELISA assay. Refer to Table 1 in main text for the composition of each formulation. TH – 0.01% w/v thimerosal.


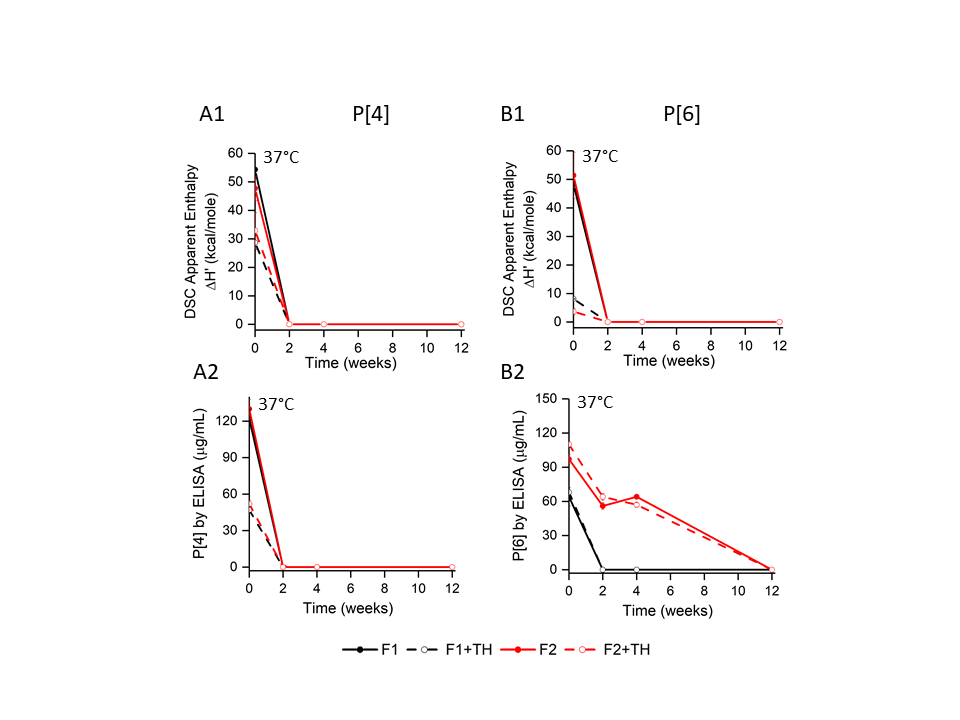


**Supplementary Figure S6.** SDS-PAGE analysis of P[4] antigen under non-reducing and reducing conditions after forced desorption from Alhydrogel adjuvant at different time points during 12 week storage at different temperatures. TH – 0.01% w/v thimerosal. Refer to Table 1 in main text for the composition of each formulation.


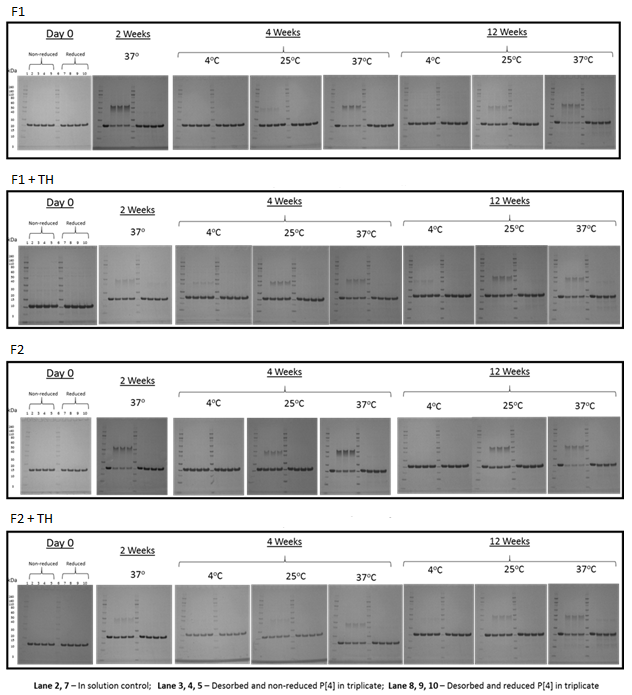


**Supplementary Figure S7.** SDS-PAGE analysis of P[6] antigen under non-reducing and reducing conditions after forced desorption from Alhydrogel adjuvant at different time points during 12 week storage at different temperatures. Refer to Table 1 in main text for the composition of each formulation. TH – 0.01% w/v thimerosal.


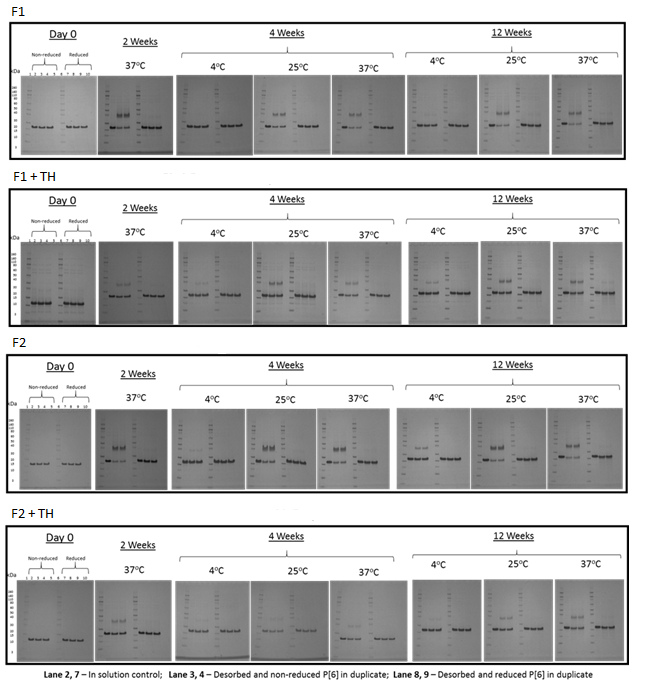


**Supplementary Figure S8.** Non-native disulfide formation at Cys^172^ and deamidation at Asn^7^ of the monovalent P[4] and P[6] antigens bound to Alhydrogel adjuvant during 12 weeks of storage at different temperatures in four different formulations. (A1, B1) Percent monomer/native-like species from non-reduced SDS-PAGE analysis, and (A2, B2) relative deamidation of Asn^7^ over 12 weeks of storage in formulations F2 and F2+TH as determined from LC-MS peptide mapping analysis. Error bars represent 1 SD from triplicate and duplicate vials for P[4] and P[6], respectively. Refer to Table 1 in main text for the composition of F2. TH – 0.01% w/v thimerosal.


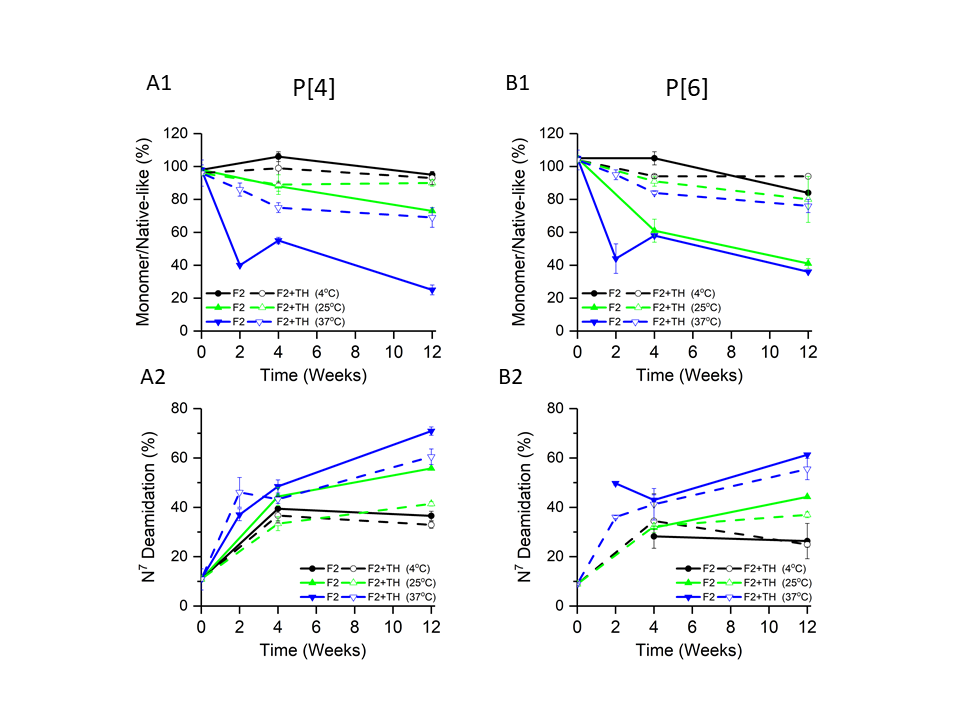


**Supplementary Figure S9.** Deamidation analysis of Asn^90^ of the P[6] antigen bound to aluminum adjuvant Alhydrogel during 12 weeks of storage at different temperatures in four different formulations from LC-MS peptide mapping analysis. Error bars represent 1 SD from duplicate vials. Refer to Table 1 in main text for the composition of each formulation. TH – 0.01% w/v thimerosal.


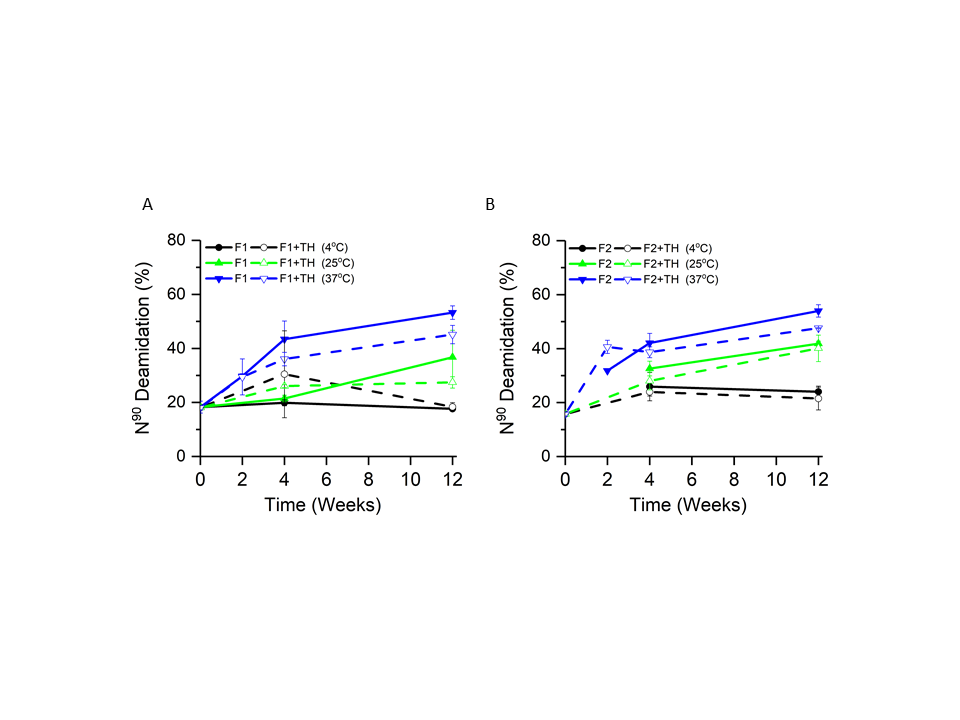


**Supplementary Figure S10.** Representative DSC thermograms of P[8] antigen bound to AH in F1 in the presence of 1.0% 2-PE over 12 weeks of storage at (A) 4°C and (b) 37°C. Refer to Figure 9 in main text for comparison of apparent enthalpy of unfolding (ΔH’) values of AH bound P[8] in three different formulations: F1, F1 + 0.01% thimerosal, and F1 + 1.0% 2-PE.

**
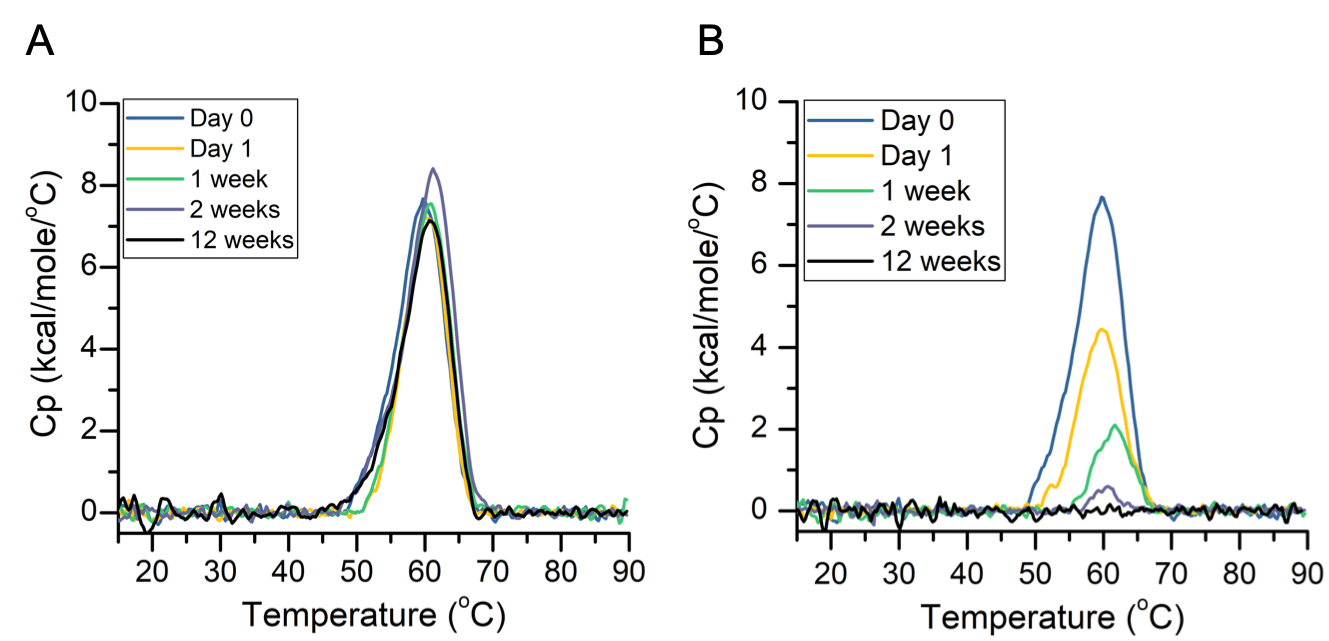
**
